# Wnt5a Suppresses Tumor Formation and Redirects Tumor Phenotype in MMTV-Wnt1 Tumors

**DOI:** 10.1371/journal.pone.0113247

**Published:** 2014-11-17

**Authors:** Stephanie L. Easter, Elizabeth H. Mitchell, Sarah E. Baxley, Renee Desmond, Andra R. Frost, Rosa Serra

**Affiliations:** 1 Department of Cell, Developmental, and Integrative Biology, University of Alabama at Birmingham, Birmingham, AL, 35294, United States of America; 2 Department of Medicine, Biostatistics and Bioinformatics Unit, Comprehensive Cancer Center, University of Alabama at Birmingham, Birmingham, AL, 35294, United States of America; 3 Department of Pathology, Division of Anatomic Pathology, University of Alabama at Birmingham, Birmingham, AL, 35294, United States of America; Baylor College of Medicine, United States of America

## Abstract

Wnt5a is a non-canonical signaling Wnt that has been implicated in tumor suppression. We previously showed that loss of Wnt5a in MMTV-PyVmT tumors resulted in a switch in tumor phenotype resulting in tumors with increased basal phenotype and high Wnt/β-catenin signaling. The object of this study was to test the hypothesis that Wnt5a can act to inhibit tumors formed by activation of Wnt/β-catenin signaling. To this end, we characterized tumor and non-tumor mammary tissue from MMTV-Wnt1 and double transgenic MMTV-Wnt1;MMTV-Wnt5a mice. Wnt5a containing mice demonstrated fewer tumors with increased latency when compared to MMTV-Wnt1 controls. Expression of markers for basal-like tumors was down-regulated in the tumors that formed in the presence of Wnt5a indicating a phenotypic switch. Reduced canonical Wnt signaling was detected in double transgenic tumors as a decrease in active β-catenin protein and a decrease in Axin2 mRNA transcript levels. In non-tumor tissues, over-expression of Wnt5a in MMTV-Wnt1 mammary glands resulted in attenuation of phenotypes normally observed in MMTV-Wnt1 glands including hyperbranching and increased progenitor and basal cell populations. Even though Wnt5a could antagonize Wnt/β-catenin signaling in primary mammary epithelial cells in culture, reduced Wnt/β-catenin signaling was not detected in non-tumor MMTV-Wnt1;Wnt5a tissue in vivo. The data demonstrate that Wnt5a suppresses tumor formation and promotes a phenotypic shift in MMTV-Wnt1 tumors.

## Introduction

Wingless-related (Wnt) proteins are a family of secreted growth factors that regulate a variety of cellular processes during development and tissue maintenance. Multiple Wnt genes are expressed in the mammary gland and they play key roles in regulating mammary gland development [1], [2]. Alterations in Wnt signaling can foster an environment favorable for the onset of breast cancer [3], [4]. Members of the Wnt family can be broadly divided into two categories: the canonical Wnt, β-catenin dependent pathway, and the non-canonical Wnt, β-catenin independent pathway [5], [6], [7]. Wnt/β-catenin signaling is associated with stimulation of cell growth and proliferation as well as cell fate specification. The non-canonical Wnt pathway can control processes involved in cell movement, motility, and polarity. It has also been suggested that specific non-canonical Wnts, including Wnt5a, can act by directly antagonizing canonical, β-catenin signaling [8], [9], [10], although the mechanism appears to vary among tissue types.

Multiple lines of evidence suggest that the non-canonical Wnt, Wnt5a, functions as a tumor suppressor protein. Loss of Wnt5a in invasive ductal breast carcinomas is associated with early relapse, increased metastasis, and poor survival [11], [12], [13]. In a screen of Wnt mRNA expression levels in various established human breast cancer cell lines, down-regulation of non-canonical Wnts, including Wnt5a, and increased expression of canonical signaling Wnts was correlated with a more aggressive phenotype [14]. Furthermore, reduced Wnt5a expression in cell culture systems led to cellular transformation similar to that induced by increased Wnt/β-catenin signaling and Wnt1-transformed epithelial cells regained normal morphological properties upon expression of Wnt5a [15], [16], [17], [18]. Other studies suggest that Wnt5a can promote tumor progression [19], [20], [21]; therefore, the effects of Wnt5a are likely dependent on the context. As such, direct *in vivo* demonstration of Wnt5a effects in vivo would be informative in establishing a role for Wnt5a as a tumor suppressor and in elucidating specific mechanisms of action.

Previous data from our laboratory demonstrated that loss of Wnt5a in tumors induced by MMTV-PyVmT resulted in increased tumor growth, redirection of the tumor phenotype to a more basal-like subtype, and increased Wnt/β-catenin signaling [22]. This suggested that Wnt5a could act as a tumor suppressor by redirecting the tumor phenotype via antagonism of the Wnt/β-catenin pathway. To characterize the effects of Wnt5a on tumors formed by constitutive activation of Wnt/β-catenin signaling, we crossed transgenic mice over-expressing the canonical Wnt, Wnt1 (MMTV-Wnt1), with mice over-expressing Wnt5a (MMTV-Wnt5a). In doing so, we found that Wnt5a suppresses MMTV-Wnt1-induced tumor formation and redirects the tumors that form to a less basal-like phenotype as measured by reduced expression of keratin 5 (K5) and keratin 6 (K6). In addition, the Wnt5a expressing tumors had less active β-catenin and lower levels of Axin2 mRNA. Analysis of non-tumor tissue demonstrated that expression of Wnt5a attenuated some of the effects of Wnt1 on the mammary gland including increased side branching and increased progenitor and basal cell populations. Wnt5a antagonized Wnt/β-catenin signaling in primary mammary epithelial cells from MMTV-Wnt1 mice although reduced signaling was not detected in vivo. Collectively, these data support a model in which Wnt5a inhibits tumor formation and redirects mammary tumor phenotype in MMTV-Wnt1 tumors.

## Methods

### Mice

MMTV-Wnt1 mice (B6SJL-TG(Wnt1)1Hev/J) were acquired from Jackson Laboratories [23] (Bar Harbor, Maine, USA). MMTV-Wnt5a mice were previously generated by our laboratory [24]. The previously characterized M5a3 line (C57Bl/6), which expressed the highest levels of Wnt5a of the lines generated were used for all of the experiments described here. Mice were housed in a specific pathogen free facility in micro-isolator cages with 1/8 inch hardwood chip bedding. Mice were kept at 72° F under a 12/12 hour light and dark cycle. Mice were provided water and rodent show (NIH 31) ad libitum. Enrichment was provided with iso-BLOX (Harlan laboratories). Male MMTV-Wnt1 mice were crossed to female MMTV-Wnt5a mice to obtain MMTV-Wnt1;MMTV-Wnt5a double transgenic male mice. For these crosses, the pups were placed with foster mothers since neither Wnt1 nor Wnt5a transgenic mice lactate efficiently. Male double transgenic mice were then crossed to wild-type female mice to generate MMTV-Wnt1;MMTV-Wnt5a double transgenic mice that were used for this study. Mice were PCR genotyped using DNA extracted from tail biopsy as previously described [23], [24]. For each experiment there were two groups, the control MMTV-Wnt1 group and the experimental group, which consisted of MMTV-Wnt1;MMTV-Wnt5a mice. Each animal was treated as an experimental unit.

### Ethics statement

All procedures using mice were approved by the University of Alabama at Birmingham, Institutional Animal Care and Use Committee under protocol number 08539 issued to RS.

### Epithelial cell isolation

Non-tumor and tumor tissue was digested with collagenase and hyaluronidase to generate mammary epithelial cell suspensions as described [25]. To further enrich for epithelial cells, cells were magnetically sorted using the Mouse Mammary Epithelial Cell Enrichment Kit (Stem Cell Technologies, Vancouver, BC, CA) according to the manufacturer's instructions. Protein or RNA was isolated directly from these cells as indicated.

### Cell line, primary cell, and mammosphere culture

Parental and Wnt5a conditioned media was generated according to standard AATC protocols using L-Parental cells (AATC #CRL-2648; parental conditioned media) and L-Wnt5a cells (AATC #CRL-2814; Wnt5a conditioned media). The indicated cells in culture were then treated for 24 hours with a 1-to-1 ratio of primary mammary epithelial cell (PMEC) media to parental or Wnt5a-conditioned media before protein or RNA isolation.

Mammary glands were digested with collagenase to generate mammary epithelial cell suspensions as described [25]. Primary mammary epithelial cells (PMEC) were plated at 1×10^5^ cells/cm^2^ in phenol red-free DMEM/F12 supplemented with 5% FBS, 0.5 µg/ml hydrocortisone, 100 ng/mL cholera toxin, 10 µg/mL insulin, 100 µg/ml penicillin, and 5 µg/ml streptomycin. To select for the E-cadherin negative population, cells were magnetically sorted to remove E-cadherin-positive cells using rabbit anti-E-cadherin antibody (1∶50, Cell Signaling, Beverly, MA, USA, #3195) according to standard protocols (Stem Cell Technologies, Vancouver, BC, CA). The resulting E-cadherin-negative cell population was also plated in PMEC medium.

For mammosphere cultures, mammary epithelial cells were isolated from virgin, 8 to 12-week-old mice in diestrus and were placed into mammosphere cultures as previously described [26]. Cells were plated onto 6-well ultra-low attachment cell culture plates (Corning, Kennebunk, ME, USA) at a concentration of 20,000 cells/well. Depending on the experiment, cells were either untreated or treated with 0.5 µg/ml rWnt5a (R&D Systems, Minneapolis, MN) upon plating, and grown for 7 to 10 days in culture. To conduct secondary mammosphere assays, primary mammospheres were trypsinized to generate a single cell suspension and were replated onto 6-well ultra-low attachment cell culture plates (Corning) at a concentration of 10,000 cells/well in the absence of exogenous rWnt5a. Mammospheres greater than approximately 50 microns were counted.

### Whole mount staining

The inguinal mammary glands (#4) were removed, fixed in Carnoy's solution (25% glacial acetic acid/75% EtOH) for 1 hour, and stained overnight with carmine. After graded ethanol dehydration steps and clearance in xylene, glands were mounted in high-viscosity toluene-based medium (ThermoScientific, Waltham, MA, USA). Mammary epithelium was quantified using Image J software to calculate pixels/mm^2^. Images were converted to binary, 16-bit grayscale and sharpened by subtracting the background. Thresholding was used to highlight epithelial areas to be measured, and area was measured using standard procedures.

### Histology and immunofluorescence

Mammary glands were removed and fixed with 4% paraformaldehyde (PFA) in 1× phosphate buffered saline (PBS) (PFA, Sigma-Aldrich, St. Louis, MO, USA) overnight at 4°C. Tissue was processed and embedded in paraffin. Five µm sections were deparaffinized with xylene and rehydrated through a graded series of ethanol. Hematoxylin and eosin (H&E) staining was performed according to manufacturer's instructions (Sigma-Aldrich). For immunofluorescence, sodium citrate (10 mM, pH 6) antigen retrieval was used for all antibodies. Rabbit anti-Keratin 6 (1∶1000, Covance, #PRB-169P), and rabbit anti-Ki-67 (1∶400, ThermoScientific, Waltham, MA, USA, #RM-9106-R7) were detected using biotin-labeled secondary antibodies (Vector Labs, Burlingame, CA, USA) followed by Alexa Fluro 488 streptavidin (Life Technologies, Carlsbad, CA, USA) and Cy-3 conjugated streptavidin (1∶1000, Zymed Laboratories, San Francisco, CA, USA, #43-4315). Nuclei were counterstained in 0.6 mM DAPI.

All images were taken using an Olympus BX50 microscope (Olympus, Center Valley, PA, USA), a magnafire camera (Olympus) and PictureFrame software (Olympus). To quantify staining, cells were counted using the ITCN (Image-based tool for counting nuclei) plug-in for ImageJ developed by Thomas Kuo and Jiyun Byun at the Center for Bio-image Informatics at UC Santa Barbara [27]. The plug-in can be downloaded without charge from www.bioimage.ucsb.edu/automatic-nuclei-counter-plug-in-for-imagej. Images were converted to eight-bit grayscale and inverted before using ITCN. Cell detection was performed by detecting dark peaks with the following parameters: cell width = 30, minimum distance = 15, threshold = 0. Data was analyzed using the T-test function in Excel.

### Western blot

Tissue was homogenized using a motorized homogenizer, and tissue and cells were lysed in RIPA buffer containing phosphatase (Sigma) and protease inhibitors (Roche, Indianapolis, IN, USA). 15 µg of the protein lysate was separated on either an 8% SDS-PAGE gel or an Any kD Mini-Protean TGX Gel (Biorad) and transferred to a polyvinylidene fluoride membrane (Bio-Rad) using the semi-dry transfer technique. Membranes were blocked in 5% non-fat dry milk and incubated with anti-active β-catenin (1∶1000, Millipore, Billerica, MA, USA, #05-665), anti-β-catenin (1∶1000, Cell Signaling Technology, Beverly, MA, USA, #9562), anti-Sca-1 (1∶1000, R&D Systems, Minneapolis, MN, USA, #AF1226), anti-Keratin 8 (1∶1000, Covance, Princeton, NJ, USA, #MMS-162P), anti-Keratin 6 (1∶1000, Covance, #PRB-169P), anti-Keratin 14 (1∶1000, Covance, #PRB-155P), anti-Keratin 5 (1∶500, Covance, #PRB-160P), or anti-h/m Wnt5a (1∶250, R&D Systems, #MAB645) according to standard procedures. Blots were then incubated in horseradish peroxidase-conjugated anti-mouse (1∶3000, Cell Signaling Technology), horseradish peroxidase-conjugated anti-rabbit (1∶3000, Cell Signaling Technology), horseradish peroxidase-conjugated anti-rat (1∶3000, Santa Cruz, Santa Cruz, USA), or horseradish peroxidase-conjugated anti-goat (1∶3000, Santa Cruz). To ensure equal protein loading between lanes, the level of glyceraldehyde 3-phosphate dehydrogenase (GAPDH) (1∶1000, Santa Cruz) was determined for each blot. All western blot images were taken on a ChemiDoc MP System (Biorad).

### Quantitative real-time RT-PCR

To determine relative levels of gene expression in the indicated cells or tissues, RNA was extracted using the Directzol RNA Mini-Prep Kit (Zymo, Ivine, CA, USA) for cells in culture or the RNeasy Mini Prep Kit (Qiagen, Valencia, CA, USA) for tissue samples. RNA samples were DNase-treated (Promega, Madison, WI, USA) according to standard protocols. QuantiFast SYBR Green RT-PCR Kit (Qiagen) was used to perform quantitative RT-PCR with specific primers that crossed intron boundaries (Table 1) on the LightCycler 480 Real-Time PCR System (Roche Applied Science, Indianapolis, IN, USA). Changes in gene expression levels were determined using beta-2-microglobulin (B2M) as the normalization gene in primary mammary cells in culture and glyceraldehyde 3-phosphate dehydrogenase (GAPDH) was used as the normalization gene in non-tumor and tumor tissue. Analysis of gene expression was performed using REST software, which may be downloaded at http://www.qiagen.com/Products/REST2009Software.aspx?r=8042 [28].

**Table 1:** List of quantitative RT-PCR primers.

Gene Name	Forward Primer (5′–3′)	Reverse Primer (5′–3′)
Axin2	AGCGCCAACGACAGCGAGTTA	GGCGGTGGGTTCTCGGAAAA
β2-Microglobulin	GCCGTGTGAACCATGTGACTTT	CCAAATGCGGCATCTTCAAA
Gapdh	TGTGTCCGTCGTGGATCTGA	TTGCTGTTGAAGTCGCAGGAG
Wnt5a (human)	CCGCGAGCGGGAGCGCATCCA CGCC	GCCACATCAGCCAGGTTGTACACC

### Tumor study statistical analysis

Tumor multiplicity data was analyzed in several ways: 1) the proportion of animals with or without tumors using the Fisher's exact test; and 2) the number of tumors compared with the Cochran-Armitage test for trend. Latency/time-to-first-tumor was analyzed using the LIFETEST (SAS Version 9.1; Cary, NC, USA). Significance was defined as *p*≤0.05.

## Results

### Wnt5a suppresses MMTV-Wnt1-induced tumor formation

**Figure 1:**
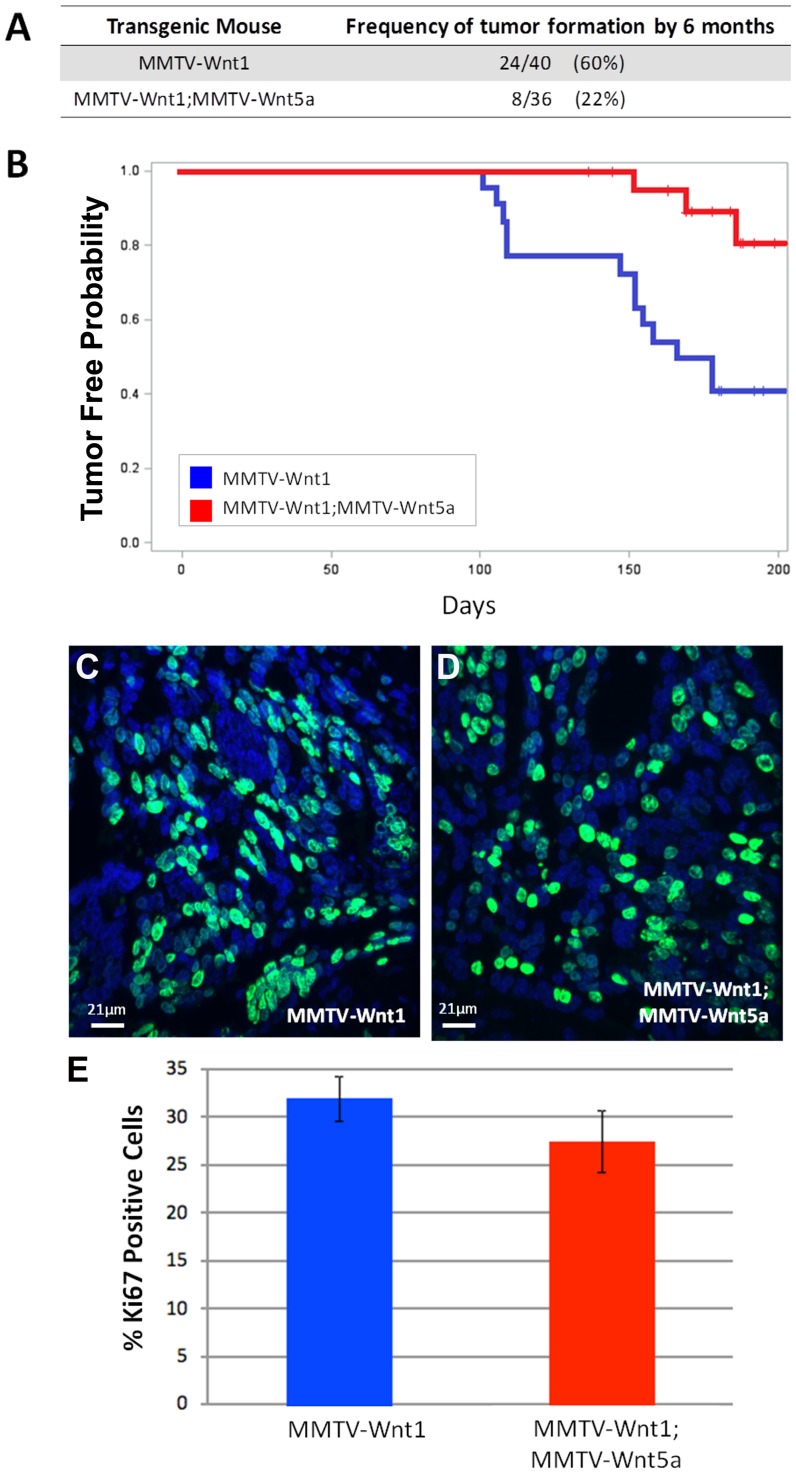
Expression of Wnt5a suppresses tumor formation and increases latency in MMTV-Wnt1 mice. (A) Frequency of tumor formation in 6-month-old MMTV-Wnt1 and MMTV-Wnt1;MMTV-Wnt5a mice (*P*-value = 0.001). (B) Kaplan-Meier-type curve to measure tumor latency. MMTV-Wnt1;MMTV-Wnt5a mice display a 6- to 7-week delay in tumor onset (*P*-value = 0.0045; n = 22 MMTV-Wnt1, n = 25 MMTV-Wnt1;MMTV-Wnt5a). (C–E) *Tumor proliferation*. Sections from MMTV-Wnt1 (C) and MMTV-Wnt1;MMTV-Wnt5a (D) mammary tumors stained with anti-Ki-67 antibody (Ki-67, green; nuclei, blue). No statistical difference (T-test) of Ki-67 staining was observed (E). Values are shown as means +/− standard error (n = 6 MMTV-Wnt1 tumors, 3 fields per tumor; n = 5 MMTV-Wnt1;MMTV-Wnt5a tumors, 3 fields per tumor).

Multiples lines of clinical evidence suggest that Wnt5a, a non-canonical signaling Wnt, can act as a tumor suppressor [11], [12], [13]. We previously demonstrated that loss of Wnt5a in MMTV-PyVmT tumors resulted in a phenotypic switch resulting in tumors with a similar basal-like pathology to tumors found in MMTV- Wnt1 mice [22]. The tumors generated in the absence of Wnt5a also demonstrated an increase in Wnt/β-catenin signaling. To address the role of Wnt5a as a tumor suppressor, we characterized tumor formation and progression in MMTV-Wnt1 and MMTV-Wnt1;MMTV-Wnt5a double transgenic mice by assessing tumor incidence, latency, and growth (Figure 1). MMTV-Wnt5a mice were previously generated and characterized in our laboratory [24]. Tumor onset was denoted by the presence of a palpable mass in the mammary fat pad. Mice were sacrificed at 6 months to collect tumor samples or earlier if the mass of the tumor exceeded 10% of the mouse body weight. MMTV-Wnt1 over-expression is known to induce mammary adenocarcinomas in approximately 50% of female transgenic mice by 6 months-of-age [23]. Compared to controls, MMTV-Wnt1;MMTV-Wnt5a mice showed reduced incidence of tumor formation (Figure 1A; 60% vs 22%, *p*-value = 0.001) and a 6- to 7-week delay in earliest detection of palpable tumors (Figure 1B; n = 22 MMTV-Wnt1, n = 25 MMTV-Wnt1;MMTV-Wnt5a, *p*-value = 0.0045). To assess whether tumor growth was affected by the presence of Wnt5a, a repeated-measure-model was used to measure subcutaneous tumor volume over time. No significant differences were observed in tumor growth (n = 13 MMTV-Wnt1, 6 MMTV-Wnt1;Wnt5a, data not shown). We also determined the level of proliferation in tumors using Ki-67 immunostaining. Differences in the percentage of Ki-67 positive cells were not detected in MMTV-Wnt1 and MMTV-Wnt1;MMTV-Wnt5a tumors (Figure 1C–E, n = 6 MMTV-Wnt1 tumors, 3 fields per tumor; n = 5 MMTV-Wnt1;MMTV-Wnt5a tumors, 3 fields per tumor). These results indicate that Wnt5a is sufficient to suppress tumors induced by Wnt1.

**Figure 2:**
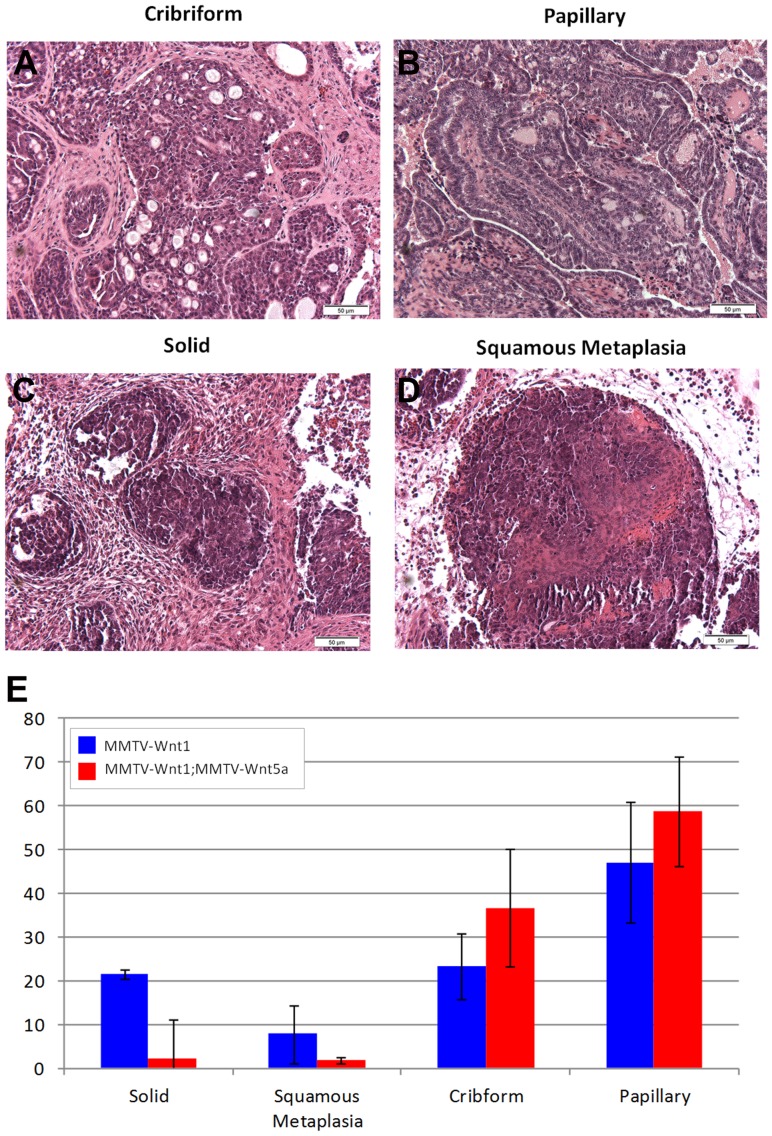
Description of histological subtypes in MMTV-Wnt1 and MMTV-Wnt1;Wnt5a tumors. Sections from tumors were stained with H&E and the percentage in each tumor of 4 different histologic patterns: cribriform (A), papillary (B), solid (C), and squamous metaplasia (D) was determined and graphed (E). Values are shown as means +/− standard error (n = 6 MMTV-Wnt1, n = 5 MMTV-Wnt1;MMTV-Wnt5a separate tumors). Both MMTV-Wnt1 and MMTV-Wnt1;MMTV-Wnt5a mice formed tumors with a mixed histology. Although a trend toward less solid and squamous tumors was observed in tumors containing Wnt5a, the changes did not reach statistical significance as determined by Mann-Whitney test for non-parametric data.

**Figure 3:**
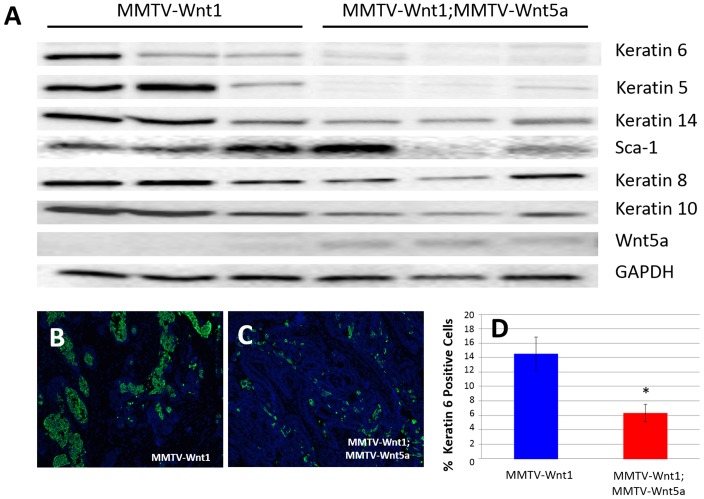
Wnt5a expressing tumors demonstrate a decrease in markers of the basal tumor subtype. (A) Western blot using protein lysates isolated from the epithelium of MMTV-Wnt1 and MMTV-1;MMTV-Wnt5a mammary tumors. The expression of molecular markers of basal and luminal tumor subtypes were compared. Keratin 6 and Keratin 5 were strongly down-regulated in Wnt5a expressing tumors. Glyceraldehyde 3-phosphate dehydrogenase (GAPDH) was used as a loading control. (B–D) *Immunostaining for K6*. Sections from MMTV-Wnt1 (B) and MMTV-1;MMTV-Wnt5a (C) tumors were stained with anti-Keratin 6 antibody using immunofluorescence (K6 = green; nuclei = blue). The percentage of cells expressing K6 was determined and graphed (D). Values are means +/− standard error (n = 6 MMTV-Wnt1, 3 fields per tumor; n = 5 MMTV-Wnt1;MMTV-Wnt5a, 3 fields per tumor). MMTV-Wnt1;MMTV-Wnt5a tumors demonstrated a significant decrease in K6-expressing cells as measured by T-test (* = p<0.05).

MMTV-Wnt1 tumors are presumed to model the basal subtype of breast cancer, which has a much poorer prognosis than luminal subtypes [29]. So next we compared the pathology of tumors from MMTV-Wnt1 and MMTV-Wnt1;MMTV-Wnt5a mice to determine if there were any obvious differences in tumor morphology. All tumors were classified as invasive adenocarcinomas, which encompassed a large degree of morphologic heterogeneity. Tumors were further characterized by the percentage of the tumor composed of 4 different histologic patterns: cribriform, papillary, solid, and squamous metaplasia (Figure 2). Cribriform (Figure 2A) and papillary patterns (Figure 2B) demonstrate more glandular or luminal differentiation than solid (Figure 2C) and squamous (Figure 2D) patterns. The degree of glandular differentiation seen on histologic examination is an important component of histologic grading systems in human breast cancers, with less glandular differentiation indicating a higher histologic grade and poorer prognosis [30]. MMTV-Wnt1;MMTV-Wnt5a tumors exhibited a trend towards a decrease in solid regions identified within tumors, suggesting a more differentiated phenotype; however, these differences did not reach the level of statistical significance (Figure 2E). To determine if Wnt5a could affect the markers associated with basal mammary cancers, we used western blot analysis to determine the level of expression of several markers including K6, K5, K14, Sca-1, K8, and keratin 10 (K10) (Figure 3A). Tumors were digested to form a single cell suspension and CD45/CD31/TER110/BP-1 containing cells were removed using magnetic sorting in order to remove stromal cells in each sample. Protein lysates were then generated from the purified tumor epithelial cells. MMTV-Wnt1;MMTV-Wnt5a tumors demonstrated less expression of K5 and K6 compared to MMTV-Wnt1 tumors (Figure 3A). It is known that K5 and K6 are markers of the basal subtype of breast cancer [31] in addition to K6 marking bipotent progenitors in non-tumor tissue [32]. To determine if reduced K6 protein was due to a decrease in the population of cells that expressed K6, we used immunostaining (Figure 3B, C). The percentage of K6 expressing cells was calculated and compared demonstrating significantly fewer K6 expressing cells in the double transgenic samples (Figure 3D; n = 6 MMTV-Wnt1 tumors, 3 fields per tumor; n = 5 MMTV-Wnt1;MMTV-Wnt5a tumors, 3 fields per tumor, *p*-value = 0.0042). Expression of K14, another marker for basal-like tumors, was more modestly reduced in Wnt5a expressing tumors compared to the MMTV-Wnt controls. Sca-1, K8, and K10, markers for luminal type cells did not show consistent differences between tumors (Figure 3A). We conclude that Wnt5a can alter the basal-like phenotype usually found in Wnt1-induced tumors.

**Figure 4:**
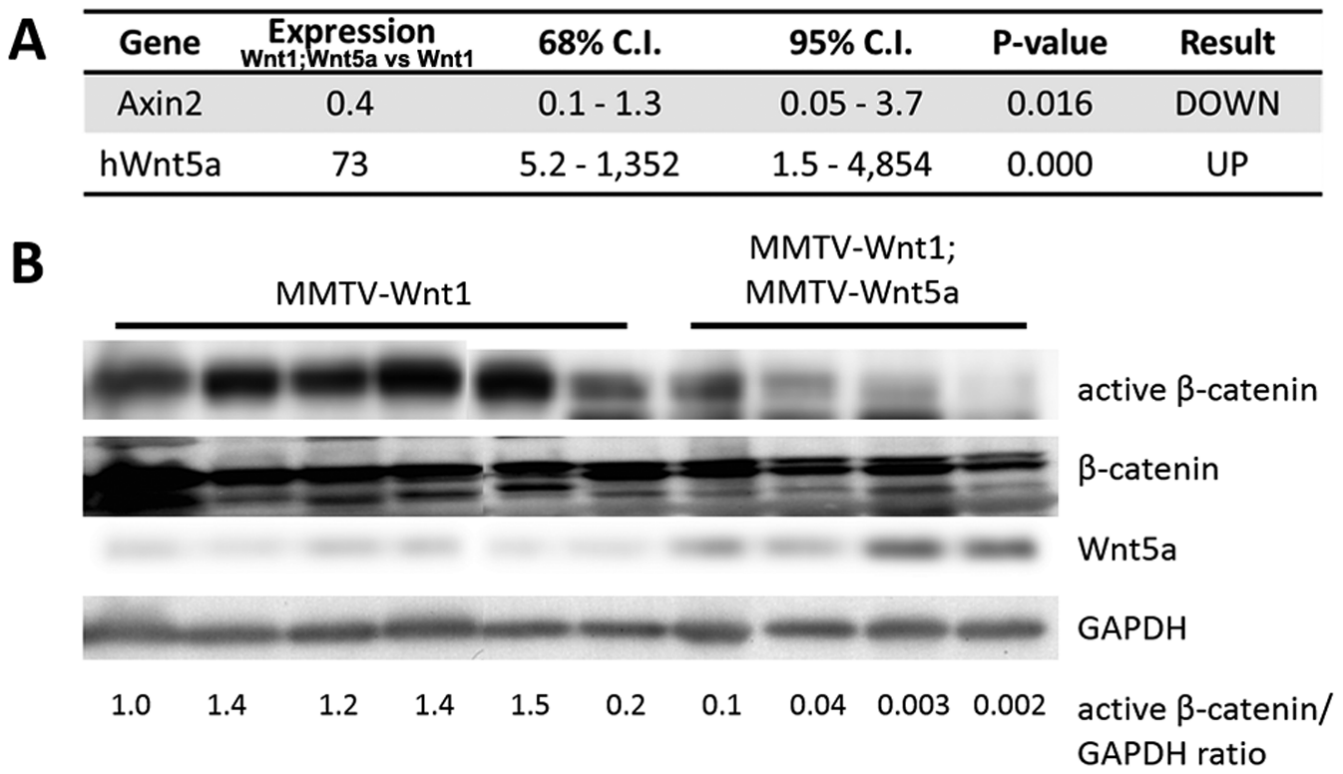
Wnt5a expressing tumors have less Wnt/β-catenin signaling than MMTV-Wnt1 tumors. (A) *Quantitative RT-PCR of Wnt/*β*-catenin target genes*. Expression of *Axin2* mRNA in MMTV-Wnt1 versus MMTV-Wnt1;MMTV-Wnt5a tumors as determined by quantitative RT-PCR (n = 5 MMTV-Wnt1, n = 5 MMTV-Wnt1;MMTV-Wnt5a). Data are shown as tables obtained using REST software. *Axin2* mRNA was significantly down-regulated in MMTV-Wnt1;MMTV-Wnt5a tumors. (B) *Western blot for β-catenin protein*. Protein lysates were prepared from MMTV-Wnt1 and MMTV-Wnt1;MMTV-Wnt5a tumors. β-catenin and glyceraldehyde 3-phosphate dehydrogenase (GAPDH) were used as loading controls. The ratio of active β-catenin to β-catenin as determined by densitometic analysis is shown. MMTV-Wnt1;MMTV-Wnt5a tumors displayed decreased levels of active β-catenin compared to controls.

Since Wnt5a was able to suppress tumor formation and alter the subtype of MMTV-Wnt1 tumors, we tested the hypothesis that Wnt5a expression in these tumors would also result in reduced Wnt/β-catenin signaling. To test this hypothesis, we compared the mRNA levels of Axin2 mRNA in MMTV-Wnt1 and MMTV-Wnt1;MMTV-Wnt5a tumors by quantitative RT-PCR (Figure 4A). Wnt5a tumors showed a significant down-regulation of Axin2 mRNA (n = 5 MMTV-Wnt1, n = 5 MMTV-Wnt1;MMTV-Wnt5a, p<0.05, REST). Next, we determined if levels of active β-catenin were altered in the Wnt5a expressing tumors. Down-regulation of active β-catenin protein was observed in MMTV-Wnt1;MMTV-Wnt5a tumors when compared to controls (Figure 4B). The data suggest expression of Wnt5a results in MMTV-Wnt1 tumors with reduced Wnt/β-catenin signaling.

### Wnt5a attenuates phenotypes observed in MMTV-Wnt1 mammary glands

**Figure 5:**
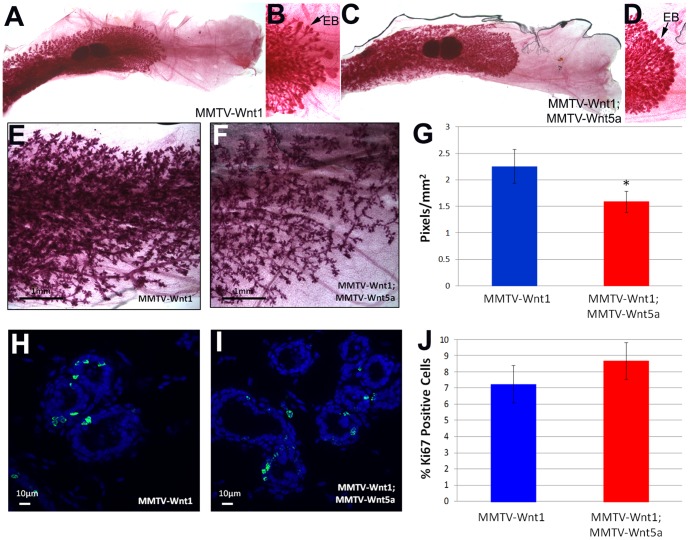
Wnt5a does not affect early development in MMTV-Wnt1 mammary glands. (A–D) *Whole mount staining, 8 weeks*. Mammary glands from 8 week old MMTV-Wnt1 (A, B) and MMTV-Wnt1;MMTV-Wnt5a (C, D) mice were stained with carmine (n = 2 each). Delay in ductal extension through the fat pad was not detected and terminal end buds (arrow, EB) were intact in Wnt5a containing glands. (E–G) *Whole mount staining, 6 months*. Mammary glands from 6-month-old, virgin MMTV-Wnt1 (E) and MMTV-Wnt1;MMTV-Wnt5a (F) mice were stained with carmine. Quantification of the area of mammary epithelium in images from the carmine stained glands (G) demonstrated a significant decrease in MMTV-Wnt1;MMTV-Wnt5a ducts compared to MMTV-Wnt1 controls. Values are shown as means +/− standard error (n = 8 MMTV-Wnt1; n = 6 MMTV-Wnt1;MMTV-Wnt5a, T-test *p-value = 0.05). (H–J) *Ki-67 staining*. Sections from MMTV-Wnt1 (H) and MMTVWnt1;MMTV-Wnt5a (I) mammary glands were immunostained using an anti-Ki-67 antibody (Ki-67, green; nuclei, blue). No statistical difference in the percentage of Ki-67 positive cells was observed (J). All values are shown as means +/− standard error (n = 6 MMTV-Wnt1, 3 fields per mammary gland; n = 5 MMTV-Wnt1;MMTV-Wnt5a, 3 fields per mammary gland).

To begin to understand the mechanism of how Wnt5a inhibited tumor formation, we compared non-tumor tissue from MMTV-Wnt1 and MMTV-Wnt1;Wnt5a mice. Mammary glands from MMTV-Wnt1 mice demonstrate increased branching, precocious alveolar development, and diffuse lobular hyperplasia [23]. The phenotype of MMTV-Wnt1 mice is due to activation of Wnt/β-catenin signaling in basal cells of the mammary gland [33]. We investigated the ability of Wnt5a, a non-canonical signaling Wnt, to inhibit some of these phenotypes by crossing MMTV-Wnt1 mice to the MMTV-Wnt5a mice. We did not detect significant differences in MMTV-Wnt1 and MMTV-Wnt1;Wnt5a glands at 8 weeks of age (Figure 5A–D). There was no delay in extension of the ducts through the fat pad and end buds were intact in the Wnt5a containing glands (Figure 5A–D). As expected, whole mount preparations of adult (6 month old) MMTV-Wnt1 mammary glands showed inappropriate lobulo-alveolar development with increased ductal side branching (Figure 5E). There appeared to be less side branching and alveolar development in MMTV-Wnt1;MMTV-Wnt5a mice at this age (Figure 5F). To quantify the observed difference, the area of epithelium was measured as pixels in images of whole mount stains. Quantification indicated that MMTV-Wnt1;MMTV-Wnt5a possessed a significantly reduced amount of epithelium within mammary glands relative to MMTV-Wnt1 controls (Figure 5G; n = 8 MMTV-Wnt1; n = 6 MMTV-Wnt1;MMTV-Wnt5a, *p*-value = 0.045, T-test), suggesting that Wnt5a attenuated excessive side branching in 6 month old Wnt1 expressing glands. Next, we used Ki-67 immunostaining to compare proliferation in MMTV-Wnt1 and double transgenic glands (Figure 5H–J). Ki-67 is commonly used as a marker of proliferation as it is present in cells going through the cell cycle and absent from quiescent cells [34]. Differences in the levels of proliferation in MMTV-Wnt1 and MMTV-Wnt1;MMTV-Wnt5a glands were not detected (Figure 5J, n = 6 MMTV-Wnt1, 3 fields per mammary gland; n = 5 MMTV-Wnt1;MMTV-Wnt5a, 3 fields per mammary gland, T-test), indicating that reduced side branching was not simply due to reduced overall cellular proliferation.

**Figure 6:**
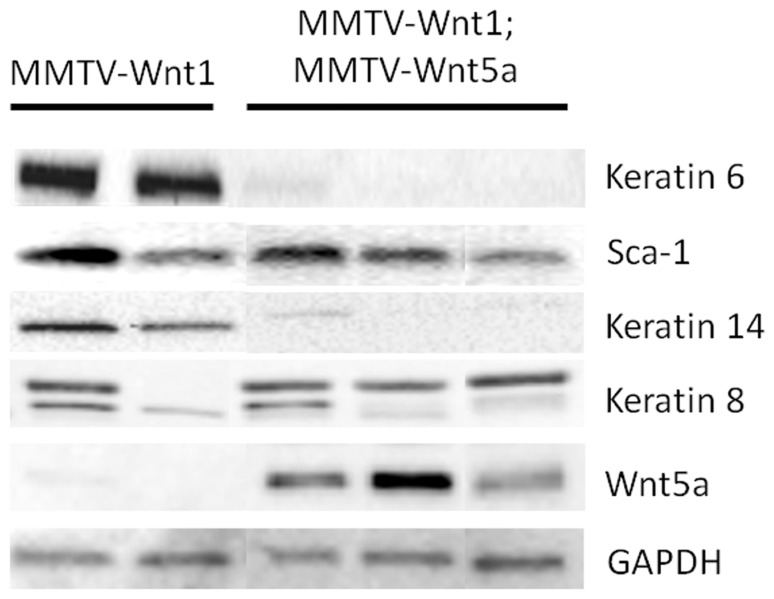
Ectopic expression of Wnt5a results in low expression of K6 and K14. Expression of molecular markers for basal and luminal progenitors in MMTV-Wnt1 and MMTV-Wnt1;MMTV-Wnt5a mammary glands was compared by western blot. Glyceraldehyde 3-phosphate dehydrogenase (GAPDH) was used as a loading control. Each lane contains protein isolated from a separate mouse.

MMTV-Wnt1 mammary glands have been shown to contain high levels of mammary progenitor cells compared to wild-type mice [33], [35], [36], [37], which suggests that activation of the Wnt/β-catenin signaling pathway results in inhibition of differentiation and expansion of undifferentiated cells in the mammary gland [26], [38], [39], [40], [41]. Increased expression of K6, a marker for bipotent progenitor cells, has been observed in MMTV-Wnt1 mammary glands [32], [36]. Moreover, MMTV-Wnt1 glands contain a higher ratio of keratin 14 (K14), a marker of basal cells [42], [43], to keratin 8 (K8), a marker of luminal cells [44], when compared to wild-type controls [33]. To evaluate whether Wnt5a could alter these Wnt1-induced effects, we used western blot analysis to assess the expression levels of these proteins in addition to Sca-1, a marker of luminal progenitor cells that is also increased in MMTV-Wnt1 tissue [45]. MMTV-Wnt1;MMTV-Wnt5a mammary tissue demonstrated reduced expression of K6 and K14 relative to controls (Figure 6), indicating Wnt5a can prevent the increase in progenitor and basal cells seen in Wnt1 over-expressing glands. In contrast, Sca-1 levels were not dramatically affected in the presence of Wnt5a, suggesting Wnt5a does not affect this luminal progenitor population in the MMTV-Wnt1 mice.

**Figure 7:**
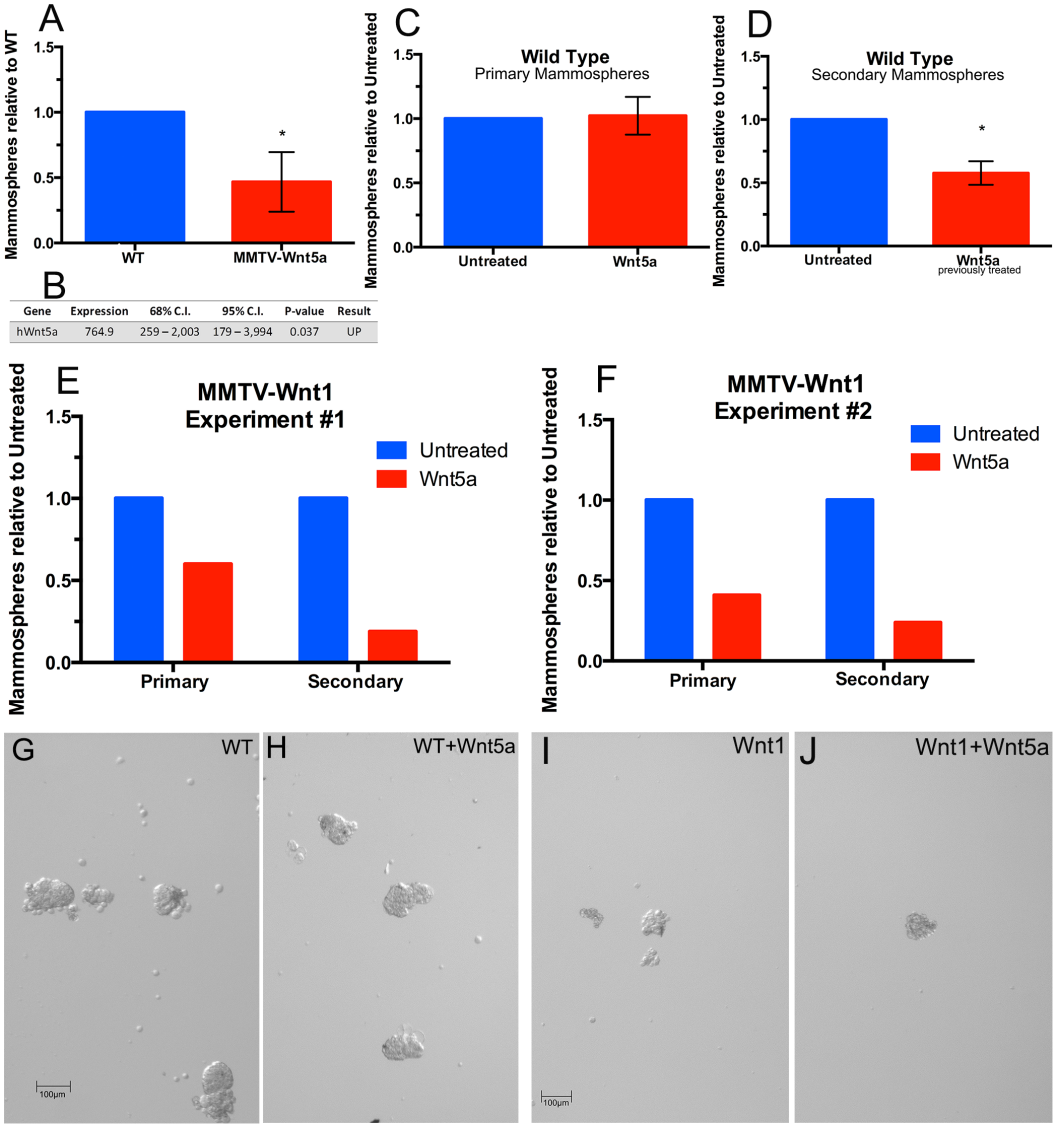
Wnt5a inhibits mammosphere formation. (A) Mammosphere cultures were derived from wild-type (WT) and MMTV-Wnt5a mammary epithelial cells. Fewer mammospheres formed in cultures derived from MMTV-Wnt5a mice when compared to those from WT controls. Values are shown relative to the WT control as means +/− standard deviation (cultures from n = 4 WT, n = 4 MMTV-Wnt5a separate mice, T-test, *p-value<0.05). (B) Over-expression of the human Wnt5a transgene in cultures derived from MMTV-Wnt5a mice was verified by quantitative RT-PCR. Expression = fold over WT control after normalization. (C, D) Primary mammosphere cultures from WT mice were grown in the presence or absence of Wnt5a (0.5 µg/ml). Differences in the number of primary mammospheres after 10 days were not detected (C; n = 4 separate experiments). The primary mammospheres were dissociated to form secondary mammospheres, which were not treated with additional Wnt5a (D). Fewer mammospheres grew in the previously Wnt5a-treated cells. Values are shown relative to the untreated controls as means +/− standard deviation (n = 4 separate experiments, *p-value<0.05). (E, F) Primary mammosphere cultures from MMTV-Wnt1 mice were grown in the presence or absence of Wnt5a (0.5 µg/ml). The primary mammospheres were dissociated to form secondary mammospheres, which were not treated with additional Wnt5a. Secondary mammospheres were reduced in previously Wnt5a treated cultures in two separate experiments shown in E and F. (G–J) Mammosphere images. Representative untreated (G, I) and previously Wnt5a treated (H, J) secondary mammospheres grown for 10 (wild type) or 7 days (MMTV-Wnt1) days in culture from wild type (G, H) and MMTV-Wnt1 (H, J) mice are shown.

To determine if Wnt5a affected a more primitive stem/progenitor cell population in the mammary gland, we used the mammosphere assay [26]. In this assay, a single cell suspension of mammary epithelial cells is plated onto low-attachment plates in a defined medium causing differentiated cells to die through a process called anoikis. The anchorage-independent growth and self-renewal properties of these progenitor cells enable their survival and formation of cell clusters called mammospheres. Each mammosphere is thought to represent a stem or early progenitor cell present in the initial population [26], [46], [47], [48]. First, we compared mammosphere formation in epithelial cells from wild-type and MMTV-Wnt5a glands (Figure 7A). 20,000 cells were plated into each well and the number of spheres over 50 µm was counted after growth in culture for 10 days. We observed that the average number of mammospheres that formed was significantly reduced in MMTV-Wnt5a cultures (Figure 7, n = 4 separate experiments, T-test p<0.05) and confirmed over-expression of the human Wnt5a transgene in the mammosphere cultures using quantitative RT-PCR (Figure 7B). Next, we isolated mammary epithelial cells from wild-type mice and plated them in mammosphere culture in the presence or absence of recombinant Wnt5a (0.5 µg/ml). After 10 days in culture, differences in the numbers of mammospheres grown in the presence or absence of Wnt5a were not detected (Figure 7C). Primary mammospheres were then dissociated and plated at 10,000 cells per well into secondary mammosphere cultures. Additional Wnt5a was not added to the secondary cultures so that we could measure changes in the levels of mammosphere forming cells that were present after Wnt5a treatment without confounding effects of continuous Wnt5a signaling. After 10 days of secondary culture, there was a significant decrease in the number of mammospheres formed in cultures that had been previously treated with Wnt5a (Figure 7D, n = 4 separate experiments, T-test p<0.05). Together, the results indicate that Wnt5a can limit the mammosphere forming progenitor populations in wild type mammary epithelium. Next, we tested if Wnt5a could inhibit mammosphere formation in cells from MMTV-Wnt1 mice. Similar to the experiment described above, mammary epithelial cells from MMTV-Wnt1 mice were placed in primary mammosphere culture in the presence or absence of recombinant Wnt5a (0.5 µg/ml). After 10 days in culture, there was a decrease in the total number of mammospheres that grew out in the presence of Wnt5a in two separate experiments (Figure 7 E, F). Secondary mammospheres were plated as described above. Similar to what was observed using cells from wild type mice, there was a decrease in the number of secondary mammospheres that grew out from the Wnt5a treated MMTV-Wnt1 cells after seven days in culture compared to the untreated MMTV-Wnt1 controls in both experiments (Figure 7 E, F). We did not detect differences in the size of secondary mammospheres in untreated or previously Wnt5a treated cultures (Figure 7G–J). The results indicated that Wnt5a can limit mammosphere forming progenitor cells from wild type as well as MMTV-Wnt1 mice.

**Figure 8:**
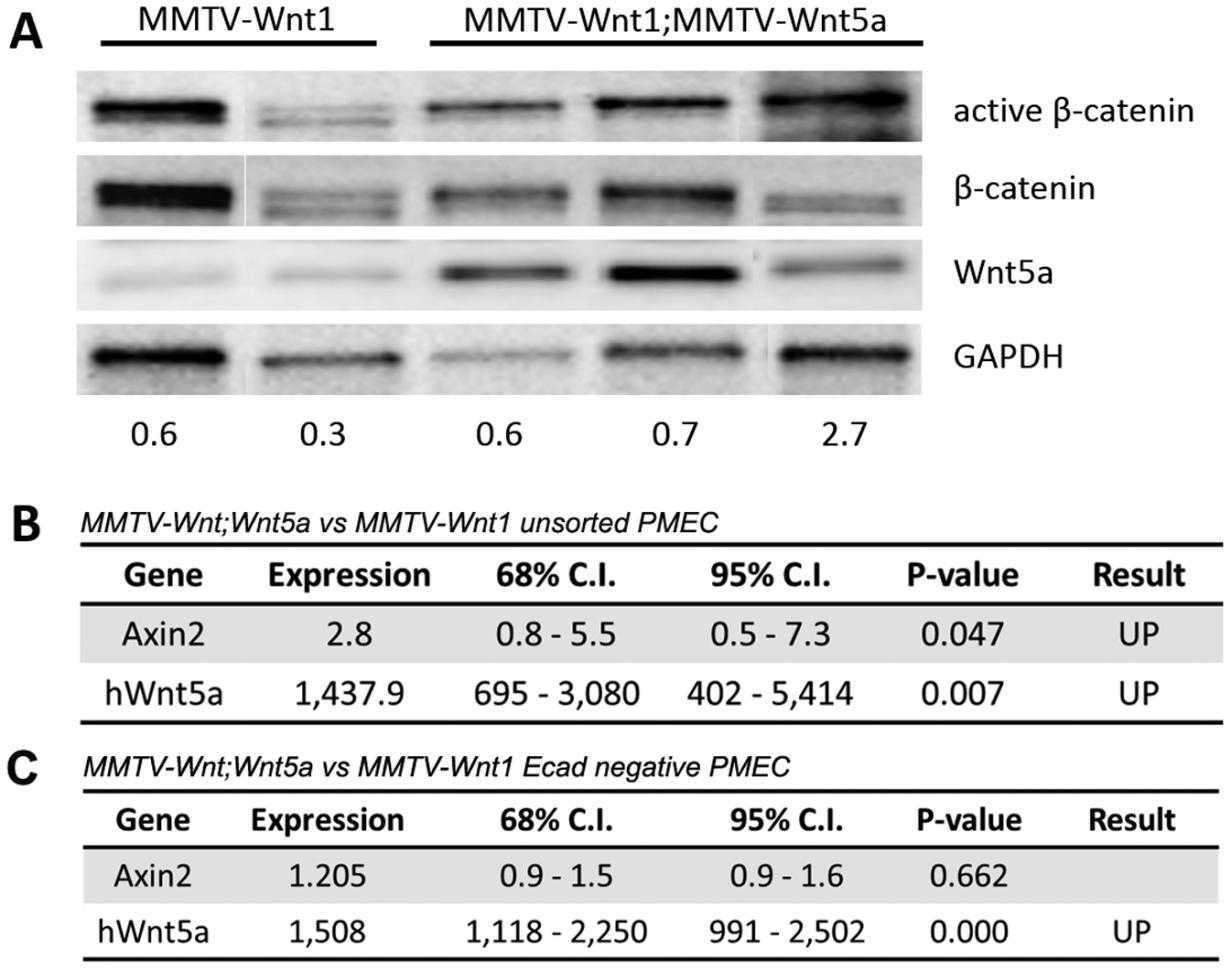
Wnt/β-catenin signaling is not down-regulated in Wnt5a expressing MMTV-Wnt1 glands. (A) *Western blot analysis of β-catenin protein in MMTV-Wnt1mammary gland*. The level of active β-catenin was compared in protein lysates from MMTV-Wnt1 and MMTV-Wnt1;MMTV-Wnt5a mammary glands. Total β-catenin and glyceraldehyde 3-phosphate dehydrogenase (GAPDH) were used as loading controls. The ratio of active β-catenin-to-β-catenin is shown at the bottom on the gel. Each lane represents a sample from a different mouse. (B) *Quantitative RT-PCR of Axin2, a Wnt/β-catenin target gene, in unsorted primary mammary epithelial cells*. Expression of *Axin2 and hWnt5a m*RNA in unsorted PMECs from MMTV-Wnt1;MMTV-Wnt5a vs. MMTV-Wnt1 mammary glands was determined by quantitative RT-PCR (n = 12 MMTV-Wnt1, n = 11 MMTV-Wnt1;MMTV-Wnt5a separate mice). Data are shown as tables obtained using REST analysis software. Expression = fold difference in MMTV-Wnt1;Wnt5a relative to MMTV-Wnt1 controls after normalization to Gapdh. (C) *Quantitative RT-PCR of Axin2 in E-cadherin negative primary mammary epithelial cells*. Expression of *Axin2* and *hWnt5a* mRNA in E-cadherin (Ecad) negative PMECs from MMTV-Wnt1;MMTV-Wnt5a vs. MMTV-Wnt1 mammary glands was determined by quantitative RT-PCR (n = 2 MMTV-Wnt1, n = 2 MMTV-Wnt1;MMTV-Wnt5a separate mice). Data are shown as tables obtained using REST analysis software. Expression = fold difference in MMTV-Wnt1;Wnt5a relative to MMTV-Wnt1 controls after normalization to Gapdh.

**Figure 9:**
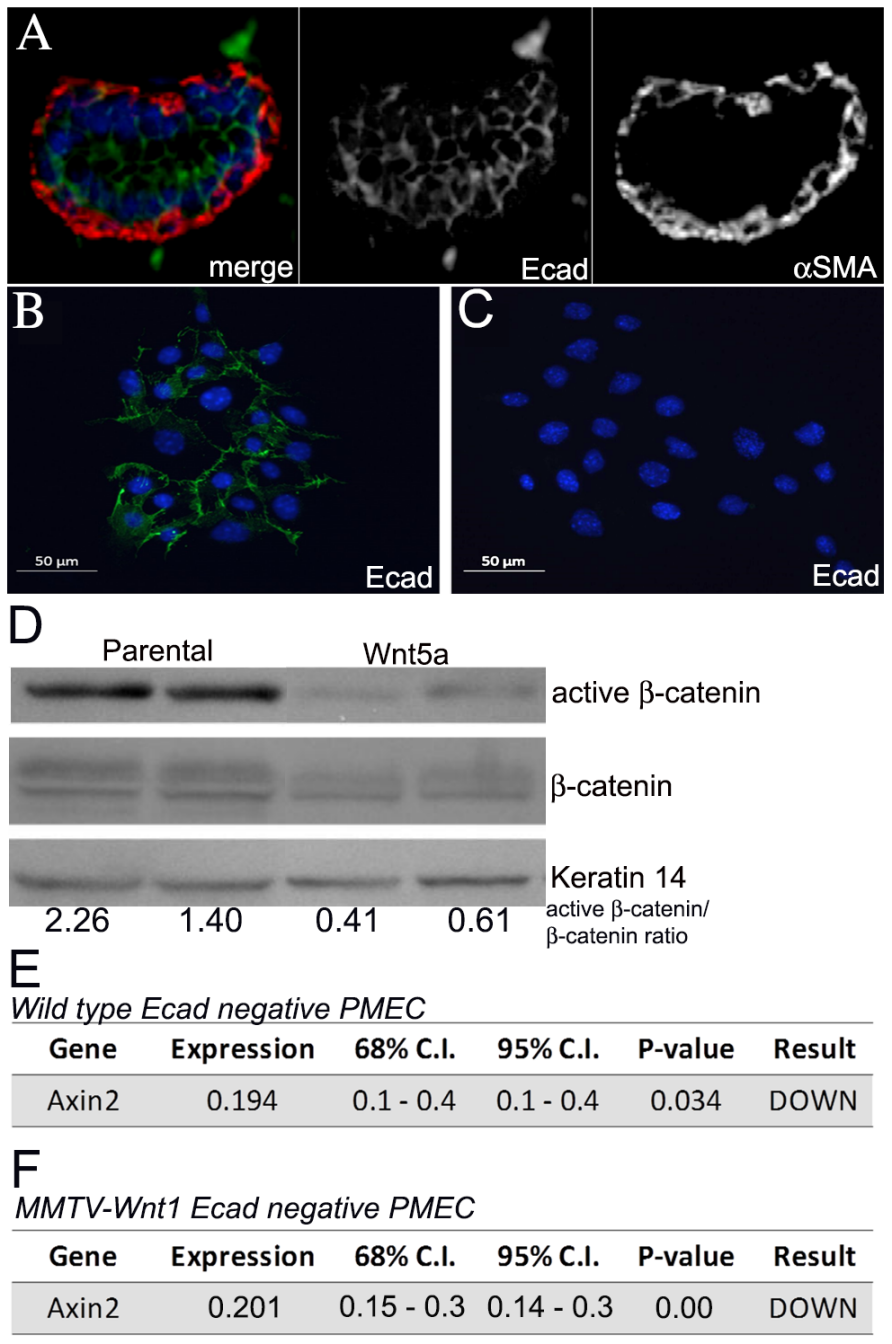
Wnt5a inhibits Wnt/β-catenin signaling in E-cadherin negative wild type and MMTV-Wnt1 cells. (A) *Immunostaining for E-cadherin and α-smooth muscle actin*. Sections from MMTV-Wnt1 mammary glands were stained with both anti- E-cadherin (Ecad, green) and anti- α-smooth muscle actin (αSMA, red) antibodies. The merged image of both red and green channels is shown (left) as well as separate channels for Ecad (middle) and αSMA (right). No overlap between Ecad and αSMA was detected indicating Ecad is not expressed in the basal myoepithelial cells. (B–C) *Immunostaining for E-cadherin on sorted cells*. Total (B) and E-cadherin negative (C) PMECs were plated overnight and then fixed and stained with anti-E-cadherin antibody (green). Nuclei were counterstained with DAPI (blue). Sorted cells demonstrated little to no E-cadherin staining. (D) *Western blot analysis of β-catenin protein in isolated E-Cadherin-negative cells*. Western blot analysis using protein lysates prepared from E-Cadherin-negative cells that were isolated from wild type mammary glands and treated with parental or Wnt5a conditioned media for 24 hours. Keratin 14 was used as the protein loading control. The average active β-catenin/β-catenin ratios are shown for cells isolated from 2 separate mice. Active β-catenin was reduced after treatment with Wnt5a conditioned media an average of 70% in five separate experiments. (E) *Quantitative RT-PCR of Axin2, a Wnt/β-catenin target gene, in wild type E-Cadherin-negative cells*. Expression of Axin2 mRNA in E-Cadherin-negative cells after treatment with parental or Wnt5a conditioned media as determined by quantitative RT-PCR (n = 3 separate experiments). Data are shown as tables obtained using REST software. *Axin2*, a Wnt/β-catenin target gene, was down-regulated after treatment with Wnt5a conditioned media. Expression is the fold difference in Wnt5a treated versus parental media treated after normalization. Gapdh mRNA levels were used as normalization controls. (F) *Quantitative RT-PCR of Axin2 in E-Cadherin-negative cells from MMTV-Wnt1 mice*. Expression of Axin2 mRNA in E-Cadherin-negative cells after treatment with parental or Wnt5a conditioned media as determined by quantitative RT-PCR (n = 2 separate experiments). Data are shown as tables obtained using REST software. *Axin2*, a Wnt/β-catenin target gene, was down-regulated after treatment with Wnt5a conditioned media. Expression is the fold difference in Wnt5a treated versus parental media treated after normalization to Gapdh.

To determine whether the effects of Wnt5a *in vivo* correlated with alterations in Wnt/β-catenin signaling, the levels of active β-catenin protein and the Wnt target gene, Axin2, were compared in MMTV-Wnt1 and MMTV-Wnt1;MMTV-Wnt5a mammary glands (Figure 8). We did not detect down-regulation in the ratio of active β-catenin-to-total β-catenin in lysates from whole glands expressing Wnt5a relative to controls (Figure 8A). Furthermore, the levels of Axin2 mRNA were increased 2.8-fold (n = 12 MMTV-Wnt1, n = 11 MMTV-Wnt1;MMTV-Wnt5a *p*-value<0.05, REST; Figure 8B) in double transgenic mice, suggesting an increase in overall Wnt/β-catenin signaling in the presence of Wnt5a *in vivo*. Since recent evidence indicates that Wnt/β-catenin signaling is normally restricted to the basal cell population in the mammary gland, we wanted to determine if Wnt5a could antagonize β-catenin signaling in basal cells [33], [49], [50], [51]. Basal cells in the mammary gland primarily express P-Cadherin whereas luminal cells preferentially express E-cadherin (Figure 9A) and [51], [52], [53] so we negatively selected for basal cells by removing E-cadherin expressing cells from PMEC isolated from wild type mice using magnetic sorting. Selection was confirmed by plating unsorted and negatively selected PMEC overnight followed by immunostaining for E-cadherin (Figure 9B,C). E-cadherin (green) could be detected at cell junctions in the unsorted cells (Figure 9B) whereas little to no E-cadherin staining was observed in the negatively selected cultures (Figure 9C) confirming the procedure had worked. E-cadherin-negative cultures were then incubated overnight with parental or Wnt5a conditioned media. Cells grown in the presence of Wnt5a conditioned media demonstrated about 70% less active β-catenin protein levels when compared to cells grown in the presence of the control, parental conditioned media (Figure 9D; n = 5, mean active β-catenin to total β-catenin ratio 1 vs 0.29, p = 0.0098, T-Test). Commensurate with this finding, a 5-fold decrease in the mRNA expression of the Wnt/β-catenin target gene, Axin2, was observed (Figure 9E; n = 4, p<0.05, REST). Together these data support the conclusion that Wnt5a can directly antagonize Wnt/β-catenin signaling in a specific population (E-cadherin negative) of mammary epithelial cells from wild type mice. Next, we tested whether or not Wnt5a could antagonize Wnt/β-catenin signaling in E-cadherin negative cells isolated from MMTV-Wnt1 mammary glands. E-cadherin negative cells isolated from MMTV-Wnt1 mice were treated with parental and Wnt5a conditioned media and Axin2 mRNA levels were measured using real time RT-PCR. Similar to what was observed using cells from wild type mice, Axin2 mRNA was significantly down-regulated by Wn5a in the cultures derived from MMTV-Wnt1 mice (Figure 9F; n = 2, p<0.05, REST). The results indicate that Wnt5a is capable of antagonizing Wnt/β-catenin signaling in the E-cadherin negative population. Since we were not able to detect reduced Wnt/β-catenin signaling in lysates from the unsorted Wnt5a expressing mammary glands (Figure 8A,B), we investigated if Wnt/β-catenin signaling was altered in the E-cadherin negative population in MMTV-Wnt1;Wnt5a mice relative to MMTV-Wnt1 mice. Expression of Axin2 was comparable in E-cadherin negative cells from Wnt1 and Wnt1;Wnt5a glands (Figure 8C). Even though Wnt5a was capable of antagonizing canonical Wnt signaling, we were not able detect reduced canonical Wnt signaling by Wnt5a in the non-tumor tissue in vivo.

## Discussion

Previously, we showed that the absence of Wnt5a, a non-canonical signaling Wnt, in MMTV-PyVmT tumors led to increased tumor growth, a change in tumor phenotype to a more basal subtype, and increased canonical Wnt/β-catenin signaling [22]. The work presented here extends these findings by demonstrating that ectopic expression of Wnt5a can attenuate hyperbranching and the increase in K6 and K14 expressing cells observed in MMTV-Wnt1 mammary glands, which have constituative activation of Wnt/β-catenin signaling. In addition, Wnt5a suppressed tumor formation in MMTV-Wnt1 mice and redirected the phenotype of tumors that formed to a less basal-like subtype as indicated by a decrease in K5 and K6 expression. Wnt/β-catenin signaling, as measured by activated β-catenin and expression of Axin2, was reduced in Wnt5a expressing tumors relative to the MMTV-Wnt1 controls. In contrast, we did not detect reduced Wnt/β-catenin signaling in lysates from Wnt5a expressing mammary glands although we showed that Wnt5a is capable of directly inhibiting canonical Wnt signaling in primary mammary epithelial cells in culture.

MMTV-Wnt1 mammary glands demonstrate increased CD24^med^CD49f^hi^ stem cells, mammosphere-forming progenitors, K6 expressing bipotent progenitor cells, Sca-1-positive luminal progenitors, and K14 expressing basal cells relative to normal mouse mammary gland [33], [35], [36], [37], [54], [55]. In this study, we show that Wnt5a limits the outgrowth of mammospheres from primary mammary epithelial cells, which are presumed to correlate with the number of progenitors upon initial plating [26], [46], [56]. First, epithelium from MMTV-Wnt5a mice had fewer mammosphere forming cells than wild type mammary epithelium. Second, addition of exogenous Wnt5a to mammospheres cultured from either wild type or MMTV-Wnt1 mice resulted in a reduction in the formation of secondary mammospheres. The results suggest Wnt5a limits at least a subset of mammosphere-forming progenitor cells from wild type or MMTV-Wnt1 expressing glands. In contrast, a recent report showed that under different conditions Wnt5a could promote the formation of mammospheres [55]. Variables between the two reports include: the age of the mice, the concentration of Wnt5a, use of methylcellulose, and the timing of Wnt5a treatment. In the present study, cells were treated with Wnt5a while in primary culture then cells were grown in the absence of Wnt5a in the secondary culture to allow a direct measure how many mammosphere forming progenitor cells were present after exposure to Wnt5a. This avoids potential confounding effects of continuous Wnt5a treatment on differentiated cell types that form during the culture period. For example, regulation of proliferation, apoptosis, or differentiation of cells in culture over time. Reduced expression of a marker for bipotent progenitor cells, K6, and a marker for basal cells, K14, in non-tumor MMTV-Wnt1;MMTV-Wnt5a glands compared to MMTV-Wnt1 tissue was also observed. Interestingly, Sca-1 levels were not affected by expression of Wnt5a suggesting Wnt5a only targets a subset of progenitor cells. Although the precise target cell for Wnt5a is not yet clear, Wnt5a may act preferentially to reduce differentiation toward the basal cell lineage since Sca-1 expression was not altered but K14 expression was reduced in MMTV-Wnt1;MMTV-Wnt5a mammary glands.

One of the most significant findings of this study is that Wnt5a suppresses tumor formation induced by high Wnt/β-catenin signaling. Wnt5a suppressed tumor formation as indicated by reduced tumor incidence and increased latency. In tumors that eventually formed, alterations in tumor phenotype were observed. It is known that human tumors demonstrating a basal-like subtype tend have worse prognosis than those with a luminal subtype [30], and we show here that increased Wnt5a expression alters the basal-like phenotype in MMTV-Wnt1 tumors as measured by the presence of specific markers. K5, K6, and K14 are often used to characterize human breast tumors with basal-like subtype. Here, we show that Wnt5a results in reduced expression of these markers in MMTV-Wnt1 tumors. Clinical data demonstrates that K5/6 expression is an independent indicator of reduced relapse-free survival [57], [58]. Reduced expression of K5 and K6 in the MMTV-Wnt1;MMTV-Wnt5a tumors parallels evidence indicating that expression of Wnt5a in human tumors correlates with better prognosis [11], [12], [59] suggesting that the MMTV-Wnt1;MMTV-Wnt5a mouse is a good translational model. We detected only minimal differences in expression of Sca-1 and K8, markers for luminal cells, in Wnt5a expressing tumors relative to the MMTV-Wnt1 controls, suggesting Wnt5a may specifically target cells in the tumor that express or are destined to express basal-like markers.

We previously proposed that Wnt5a acted in the mammary gland by antagonizing canonical Wnt/β-catenin signaling [22]. Here we show that β-catenin signaling is reduced in tumors from MMTV-Wnt1;MMTV-Wnt5a mice. We could not formally rule out the possibility that reduced β-catenin signaling in Wnt5a expressing tumors was a consequence of alterations in tumor subtype. Since basal-like cells have higher Wnt/β-catenin signaling fewer basal-like cells would be expected to result in less active β -catenin and Axin2 in the tumors. When we looked at non-tumor tissue, we did not detect reduced β-catenin signaling in Wnt5a expressing glands despite an observed decrease in hyper-branching and alterations in the molecular markers for specific progenitor and basal cells, activities known to be mediated by canonical Wnt signaling. If Wnt5a acts on only a subset of cells it would be difficult to detect these changes in vivo. We showed that Wnt5a is capable of inhibiting Wnt/β-signaling in cultured wild type and MMTV-Wnt1 E-cadherin-negative PMECs, which represent an enriched population of basal cells. These results support the hypothesis that Wnt5a can directly antagonize canonical Wnt signaling. Nevertheless, we did not detect a reduction in Axin2 expression in the E-cadherin negative population in Wnt5a expressing glands in vivo making the mechanism of the phenotypes observed in MMTV-Wnt1;Wnt5a mice unclear. It is possible that there is not enough Wnt5a in the transgenic mice to affect β-catenin signaling; however, this seems unlikely since there is enough Wnt5a expressed to attenuate many of the known phenotypes in MMTV-Wnt1 mice. It is possible that Wnt5a antagonizes β-catenin in a not yet identified cell type and that signaling in other cell populations masks any changes mediated by Wnt5a in vivo. It is also possible that Wnt5a acts in vivo through a mechanism that is unrelated to β-catenin. In summary, the data support a tumor suppressive role for Wnt5a in the mammary gland.
